# Lymphocyte-Specific Biomarkers Associated With Preterm Birth and Bronchopulmonary Dysplasia

**DOI:** 10.3389/fimmu.2020.563473

**Published:** 2021-01-21

**Authors:** Soumyaroop Bhattacharya, Jared A. Mereness, Andrea M. Baran, Ravi S. Misra, Derick R. Peterson, Rita M. Ryan, Anne Marie Reynolds, Gloria S. Pryhuber, Thomas J. Mariani

**Affiliations:** ^1^ Division of Neonatology, Department of Pediatrics, University of Rochester, Rochester, NY, United States; ^2^ Department of Biostatistics and Computational Biology, University of Rochester, Rochester, NY, United States; ^3^ Department of Pediatrics, University at Buffalo, Buffalo, NY, United States; ^4^ Department of Pediatrics, Case Western Reserve University, Cleveland, OH, United States

**Keywords:** prematurity and respiratory outcomes program, bronchopulmonary dysplasia, RNA-sequencing, Pathway, Newborn Lung, Peripheral Blood Mononucleated cells, CD8+ lymphocytes

## Abstract

Many premature babies who are born with neonatal respiratory distress syndrome (RDS) go on to develop Bronchopulmonary Dysplasia (BPD) and later Post-Prematurity Respiratory Disease (PRD) at one year corrected age, characterized by persistent or recurrent lower respiratory tract symptoms frequently related to inflammation and viral infection. Transcriptomic profiles were generated from sorted peripheral blood CD8+ T cells of preterm and full-term infants enrolled with consent in the NHLBI Prematurity and Respiratory Outcomes Program (PROP) at the University of Rochester and the University at Buffalo. We identified outcome-related gene expression patterns following standard methods to identify markers for oxygen utilization and BPD as outcomes in extremely premature infants. We further identified predictor gene sets for BPD based on transcriptomic data adjusted for gestational age at birth (GAB). RNA-Seq analysis was completed for CD8+ T cells from 145 subjects. Among the subjects with highest risk for BPD (born at <29 weeks gestational age (GA); n=72), 501 genes were associated with oxygen utilization. In the same set of subjects, 571 genes were differentially expressed in subjects with a diagnosis of BPD and 105 genes were different in BPD subjects as defined by physiologic challenge. A set of 92 genes could predict BPD with a moderately high degree of accuracy. We consistently observed dysregulation of *TGFB, NRF2, HIPPO*, and *CD40*-associated pathways in BPD. Using gene expression data from both premature and full-term subjects (n=116), we identified a 28 gene set that predicted the PRD status with a moderately high level of accuracy, which also were involved in *TGFB* signaling. Transcriptomic data from sort-purified peripheral blood CD8+ T cells from 145 preterm and full-term infants identified sets of molecular markers of inflammation associated with independent development of BPD in extremely premature infants at high risk for the disease and of PRD among the preterm and full-term subjects.

## Introduction

Acute and chronic respiratory morbidities are common in extremely premature infants (1). Increased survival of very premature infants is leading to increasing numbers of children with chronic lung disease. Since the end of the last millennium, the rate of premature births <34 weeks of gestation have consistently increased in the United States, and in 2008 it was 12.3% (2). Among the extremely preterm infants, 20%–35% die before their discharge to home (1, 3). Prematurity-related deaths accounted for 35% of all infant deaths in 2010, more than any other single cause. Preterm birth cost the U.S. health care system more than $26 billion in 2005 (4). Among NICU survivors, approximately 40% develop Bronchopulmonary Dysplasia (BPD), a chronic lung disease of the newborn. BPD has both genetic and environmental risk factors. It is characterized by varying degrees of lung injury potentially due to required supplemental oxygen, exposure to inflammatory conditions *in utero*, and mechanical ventilation and is often associated with infection (5, 6). BPD results from abnormal repair and impaired lung development after acute lung injury. Airway function may even deteriorate during the first year of life in infants with BPD (7). A key component of BPD is persistent inflammation of the lung (8, 9). Infants with BPD are more likely to die than those without chronic lung disease, even if they survive the initial hospitalization. However, improved medical treatment plans have been developed that have led to a lower hospital mortality rate, however, the respiratory sequelae into childhood remain poorly defined (10). By developing a better understanding of the inflammatory process of infants with BPD, we could potentially identify biomarkers that relate to respiratory sequelae. Using such information, certain pathways could be targeted for drug development to improve the health of infants with BPD (11).

The NIH NHLBI Prematurity and Respiratory Outcomes Program (PROP) enrolled 835 extremely premature infants across the US and collected multiple biospecimens over time and extensive data including BPD and respiratory morbidity outcomes over the 1st year of life. PROP investigated the molecular mechanisms contributing to the risk of respiratory disease in premature neonates over the first year of life. A set of clinical and non-invasive respiratory assessments were performed, based on the respiratory status of the infant at the time of testing, and was used to predict the severity of respiratory outcomes in the first year of life. The primary goal of the PROP studies was to identify biomarkers (biochemical, physiological, and genetic) that are associated with, and thus potentially predictive of, respiratory morbidity in preterm infants up to 1-year corrected age. A validated, objective measure of pulmonary outcome at 1 year does not currently exist. In addition to the identification of markers of BPD, one of the primary outcomes in the PROP study was defined as presence or absence of Post-Prematurity Respiratory Disease (PRD) (12). In order to be classified as having PRD, infants were required to have a positive response in at least one of the four morbidity domains [(1) hospitalization for respiratory indication, (2) home respiratory support, (3) respiratory medication administration, and/or (4) respiratory symptoms without a cold] during at least two separate parental interviews conducted at 3-, 6-, 9-, and 12-months corrected age (13).

High throughput sequencing for genome-wide transcriptomic analysis, by RNA-Seq or microarrays, is an unbiased approach applied to identify biomarkers that may provide predictive value. These approaches have been proven to be powerful tools capable of biomarker discovery for various disease states including BPD. We have previously presented an analysis of lung tissue gene expression in subjects with BPD (14). Application of blood-based gene expression profiling can potentially provide novel biomarkers for diagnosis and therapeutic management of BPD. Previous studies have used whole blood–derived peripheral blood mononuclear cells (PBMC) as a means of mining for novel markers for BPD (15). PBMCs are relatively easy to obtain from whole blood and can be sorted into leukocytes, including B cells, T cells, monocytes, and natural killer cells (16). The use of peripheral blood has identified changes in CD4+ T cell populations in subjects with diagnosed with BPD (17, 18). Additional studies have identified that patterns of proinflammatory cytokines in blood from subjects with BPD were associated with the phenotype of BPD (19). Our study was funded to complete transcriptomics analyses of the CD8+ T cell population.

Emerging data suggest an important role for T lymphocytes in the pathogenesis of chronic lung disease in babies born prematurely. CD8+ T cells, TNF-α, TNF receptors, and NK cells provide protection from viral infection but also contribute to the immunopathology, by contact-dependent effector functions (perforin and FasL). IFN-γ and particularly TNF-α are thought to be primary perpetrators of T-cell-mediated lung injury (20). Furthermore, one study shows that CD8+ T cells isolated from blood of infants with BPD exhibited lower levels of surface CD62L, which is consistent with an activated phenotype (21). CD8+ T cells have shown adaptive immune insufficiency in newborn mice infected with influenza A within 1 week of birth (22). RSV infected neonatal mice recruited CD8+ T cells defective in IFN-γ production in association with mild symptoms. Re-infection as adults however resulted in limited viral replication but enhanced inflammation and T cell recruitment, including Th2 cells and eosinophils (23, 24). Depletion of CD8+ T cells (but not CD4) cells during the primary neonatal infection was protective against the adult challenge. We have previously shown that CD8+ T cells appear to play a pathogenic role in subjects with BPD, and may be associated with overall risk for lung morbidity (25). In related studies, we have observed that CD8+ T cells are increased in both mouse and human lungs exposed as neonates to hyperoxia, and have a hyper-responsive, fibrotic and destructive response to subsequent viral infection (14, 26). Further, these cells have a predominant role in direct cytotoxicity in the lung, *via* interactions with epithelial cells and as regulators of macrophage responses, as well as in general human resistance to viral infection. In a separate population of premature infants, enrolled in the PROP study, phenotyped T cells at birth, at 36 weeks of adjusted gestational age, and at 12-months corrected age, were associated with a PROP-defined respiratory morbidity at 12 months (27). The goal for the current study was to demonstrate that transcriptional profiling of CD8+ T cells, obtained from premature infants at discharge, can identify disease-related gene expression patterns informative for pathogenesis and capable of predicting risk of future respiratory distress. We hypothesized that transcriptomic analysis of sorted lymphocyte sub-populations could identify predictive markers and pathways associated with respiratory outcomes. We followed a cohort of 157 infants, ranging from 23 to 41 weeks of gestation at birth, enrolled in the Prematurity and Respiratory Outcomes Program (PROP) at the University of Rochester Medical Center and Children’s Hospital of Buffalo (12).

Here, we present a novel gene expression RNA-seq data set generated from CD8+ T cells from 145 subjects with varying levels of premature birth and report the identification of disease biomarkers for BPD and PRD. In subjects who were diagnosed with BPD, we identified pathways associated with TGFB signaling (Regulation of Epithelial-Mesenchymal Transition Pathway) and T cell activation and polarization (e.g., IL-2, IL-17, IL-4, and iCOS signaling). In subjects who developed PRD at one year of life, we also identified the TGFB pathway as being important as well as the Cell2Cell pathway (which includes genes important for CD8+ T cell activation). Given that TGFB has been identified as an important factor for controlling CD8+ T cell mediated inflammation (28–32), we provide new makers in CD8+ T cells that are associated with the BPD and PRD. Such information will be of interest to researchers who are trying to develop a better understanding of factors associated with inflammatory pediatric lung disease.

## Methods

This study aimed at generating transcriptomic profiles of CD8+ T cells from newborn human blood. This study was approved by the Institutional Review Board of University of Rochester with a Memorandum of Understanding executed with the University of Buffalo IRB. Subjects were enrolled within gestational age at birth (GAB) epochs, in order to characterize the relationship among prematurity, disease risk, and gene expression. The steps involved in the process, starting from sample collection to isolation of total RNA have been described in detail in our previous publication (16). Additional steps relevant to this study are shown in **Supplemental Figure 1**.

### Oxygen Exposure, BPD Diagnosis, and PRD

The traditional categorical approach of classifying BPD as absent or present is likely an oversimplification. Tooley (33) recommended that oxygen use at 28 days of age would identify preterm infants with BPD. Almost a decade later, Shennan and colleagues proposed that the best predictor of abnormal pulmonary outcomes among very low birth weight premature infants was the clinical use of oxygen at 36 weeks postmenstrual age (PMA) (34). A workshop convened by the National Institutes of Health (NIH) proposed severity-based diagnostic criteria for BPD (35) that included the use of oxygen for at least 28 days (not necessarily consecutive) and an assessment of respiratory support at 36 weeks PMA, recognizing that some infants breathing room air at 36 weeks PMA may have residual lung disease. The majority of infants with birth weights less than 1 kg will have a diagnosis of at least mild BPD by the Consensus Conference definition based on 28 days in oxygen (35). Given clinical variations in oxygen administration, a structured trial of room air test was developed by the NICHD Neonatal Research Network (36), the frequency of BPD among the subjects was determined using two previously published definitions: the Shennan definition (34), which defines BPD as supplemental oxygen requirement at 36 weeks PMA in infants born with birth weight (BW) less than 1,500 grams, and a physiologic definition with a room-air challenge (RAC) which defines BPD as requirement of oxygen support (>21% O_2_) for at least 28 days and a subsequent assessment at 36 weeks PMA or discharge, whichever comes first (13, 36).

NICU oxygen exposure was calculated, as previously reported, from the FIO2 recorded in the medical record once each noon for the first 14 days of life. FIO2 was corrected to Effective FiO2 for low nasal cannula flow using established tables (37). Oxygen utilization or Oxygen_AUC_ was calculated by the formula defined in Benaron and Benitz (37) using information recorded in the daily respiratory flowsheet data (FIO2, respiratory support mode, and applied airway pressure or cannula flow) through the first 28 days of life. We chose to look at Oxygen_AUC_ at 14 days of postnatal age (Oxygen_AUC14_) to include the second postnatal week to capture pulmonary deterioration as presented in BPD (38).

The infants enrolled in the study were followed up periodically for up to 12 months of age, corrected by gestational age (CGA) at birth. At the 12-month CGA follow-up visit they were assessed for persistent respiratory distress based on the frequency of hospitalization due to any kind of respiratory distress. Persistent Respiratory Distress (PRD) was diagnosed if there were positive responses in at least one of the following domains: (1) hospitalization for respiratory indication, (2) respiratory support at home, (3) respiratory medication administration, and/or (4) cough or wheeze without a cold, reported on at least two caregiver post-discharge surveys completed at 3, 6, 9, and 12 months CGA, as previously reported (13). The definitions and criteria for the different diagnoses have been listed in **Supplemental Table 1**.

### Sample Collection and RNA Isolation

An average of 2.5 ml of venous blood was collected into sodium heparin glass vacutainers from premature infants enrolled with consent in the Prematurity and Respiratory Outcomes Program (PROP), at the time of hospital discharge at the University of Rochester and the University at Buffalo and shipped to a central laboratory in Rochester. Freshly purified PBMCs were isolated by Ficoll gradient (Amersham Pharmacia Biotech # 17-1440-03) centrifugation, from the whole blood diluted 1:2 with 1x dPBS, and counted according to previously established protocols (39). In subjects with at least 8 million cells, 5 million cells were stained with antibodies to individual lymphocyte markers, and sorted on a FACSAriaII sorter at the Flow Cytometry Core facility of the University of Rochester as previously reported (16). CD3+CD8+, CD3+CD4+, CD3-CD56+ (NK), or CD3-CD19+ (B) cells were collected separately. Non-marker positive and dead cells were discarded. Sorted cells were spun into pellets, which were further lysed and frozen. The steps from collection to lysis of each sample were completed within a 24-h period in order to preserve RNA quality and integrity. Frozen lysates were thawed and RNA was extracted using Agilent Absolute RNA Microprep kit (catalog #400805), with an on-column DNase digestion, as per manufacturer recommended protocol.

### RNA-Seq and Data Generation

For the current study, RNA isolated from the sorted CD8+ T cells from 145 pre-term and full-term subjects, was used for transcriptomic profiling by RNA-seq. cDNA libraries were generated with 1 ng RNA, using the SMARter Ultra Low Amplification kit (Clonetech, Mountain, CA). cDNA quantity was determined with the Qubit Flourometer (Life Technologies, Grand Island, NY) and quality was assessed using the Agilent Bioanalyzer 2100 (Santa Clara, CA). Libraries were sequenced (single endreads) on the Illumina HiSeq2500 (Illumina, San Diego, CA) to generate 20 million reads/sample. Reads generated from the sequencer were aligned using the TopHat algorithm (40) and expression values were summarized using HTSeq (41). The data from this study has been provided in dbGAP. The dbGaP accession assigned to this study is phs001297.v1.p1.

### Normalization and Filtering

Samples were excluded based on poor read count/mapped read numbers, or if they were extreme outliers in hierarchical clustering and Principal Components Analysis (PCA). Genes were excluded if they were not consistently identified as expressed (a count of zero in over 1/3 of subjects). Subjects with high prevalence (>75%) of zero/low reads (raw count value ≤5) were excluded. Genes with high prevalence (>75%) of low counts (normalized count value ≤3) across subjects were excluded.

The subject-specific conditional upper quartile (UQ, 75^th^ percentile) among non-zero reads was computed. The subject-specific normalization factor was calculated by dividing the UQ for a given subject by the mean UQ across all subjects. The normalized gene values for a given subject were calculated by dividing the raw count values by the normalization factor for that subject. After adding 1 to all values to account for zeros, the normalized counts were log_2_ transformed.

### Selection of Univariately Differentially Expressed Genes

Differences in gene expression between subject groups was assessed by SAM-Seq (42) and Likelihood Ratio Test (LRT). SAM-Seq was used to identify genes with expression patterns significantly (FDR<0.05) associated with BPD, RAC. LRT for log (normalized RNA-Seq), adjusted for GAB *via* logistic regression, was used to identify genes with expression patterns significantly (FDR<0.05) associated with BPD in subjects born at less than 29 weeks of age. For quantitative analysis, the correlations between normalized counts and oxygen utilization at 14 days were estimated. Expression changes of the genes, identified as significantly different in BPD by the tests, were assessed in transcriptomic profiles of PBMCs obtained from infants with BPD, and age matched controls generated from an independent cohort (15), using the data available on Gene Expression Omnibus (GSE32472).

### Prediction of Bronchopulmonary Dysplasia (BPD) *via* Screened Principal Components

The following method is our minor variant of *Screened Principal Components Analysis (sPCA)* (43), where the genes were univariately screened, and those with a nominal Wilcoxon p < 0.10 were used for further analysis. The first principal component (PC1) of the genes was derived, and genes with loadings close to 0 were removed. Genes most strongly univariately associated with BPD (with or without adjusting for gestational age) were selected using a screening threshold chosen by cross-validation (CV). The first Principal Component (PC1) of the genes passing the univariate screen was constructed and a logistic regression model was fit to predict BPD as a function of PC1 (and optionally gestational age). Receiver Operating Characteristic (ROC) curves depicting sensitivity and specificity along with associated AUC were estimated without (naïve AUC) and with an outer loop of nested CV (CV-AUC).

### Prediction of Post-Prematurity Respiratory Disease (PRD) *via* Canonical Pathways

We have used canonical pathway analysis where we grouped our 13,434 genes into 1,330 biologically-relevant pathway-based gene sets of molecular signature database (mSigDB) (44), where each gene belongs to 0, 1, or more pathways. We then reduced the constituent genes in each pathway to their PC1. Genes belonging to 0 pathways were thus excluded from consideration, while CV screened logistic forward selection (or LASSO) was applied to the 1,330 pathway-based PC’s. Bivariate CV was used to simultaneously select both the threshold for univariate logistic likelihood ratio test screening of pathways (with or without adjusting for gestational age) and the final number of pathways chosen by forward selection (or the LASSO penalty). The entire procedure was then nested within an outer loop of nested CV in order to estimate performance *via* the Receiver Operating Characteristic (ROC) curve and its associated Area Under the ROC Curve (CV-AUC). This method has been described in details in our previous publication (45).

### Functional Classification

Genes identified as differentially expressed in individual comparisons were used for independent functional classification through canonical pathway analysis, and upstream regulators identification using Ingenuity Pathway Analysis (IPA; Qiagen, Carlsbad, CA). Pathways and upstream regulators were identified as significantly associated with the diagnoses by Fisher’s exact test (p < 0.05 or -log(p-value) > 1.3) as calculated by IPA.

### Quantitative Reverse Transcriptase–Polymerase Chain Reaction (qPCR)

cDNA was synthesized from 100 ng RNA using iScript cDNA synthesis kit (Biorad, HerculesCA) and quantitative reverse transcriptase–polymerase chain reaction (qPCR) was performed with a Viia7 (Applied Biosytems, Carlsbad, CA) using SYBR green chemistry as previously described (14) using noncommercial (http://pga.mgh.harvard.edu/primerbank) assays. Gene expression levels were calculated relative to GAPDH as an internal, endogenous control, according to the ddCT method.

## Results

### Subject Demographics

Peripheral blood was collected at the time of first hospital discharge, from 145 preterm and full-term infants enrolled in the NHLBI Prematurity and Respiratory Outcomes Program (PROP) at the University of Rochester and the University at Buffalo. Of the 145 subjects, 72 were extremely premature having been born at less 29 weeks of gestation. There was insufficient evidence that the distribution of race (p=0.64) or sex (p=1) differed between BPD and non-BPD subjects, while as expected gestational age at birth was lower for those with BPD (p=0.03). Similarly, among all subjects (n=130) there was insufficient evidence of any difference in race (p=0.70) or sex (p=1) by PRD status, while as expected gestational age at birth was lower for those with PRD (p=0.01; **Table 1**). The age (in days) at the time of sample collection varied by subject, and it ranged from four days to 6 months after birth, depending on the gestational age at birth. However, age was consistent in terms of corrected gestational age, which ranged between 39 to 41 weeks. Detailed diagnostic and demographic information for the individual subjects in provided in **Supplemental Table 2**.

**Table 1:** Subject demographics.

Subjects withGAB < 29 Weeks (n=72)	BPDN=34	Non BPDN=38	P value*
GAB in Weeks, mean ± SD	26.1 ± 1.4	26.9 ± 1.5	0.03
Male Sex, No. (%)	17 (50)	18 (47)	1.00
White Race No. (%)	20 (59)	20 (53)	0.64
**Demographics** **All Subjects (n=130)**	**PRD YES** **N=70**	**PRD NO** **N=46**	**PRD NA^#^** **N=14**	P value*
GAB in Weeks, mean ± SD	28.5 ± 3.3	31.1 ± 5.2	32.2 ± 4.1	0.01
Male Sex, No. (%)	34 (48)	23 (50)	10 (71)	1.00
White Race, No. (%)	43 (61)	30 (65)	9 (64)	0.7

*Fisher’s Exact or t-test P-value.

GAB, gestational age at birth; PRD NA^#^, PRD Status Not Available.

Transcriptomic profiles were generated from sorted and purified CD8+ T cells obtained from the blood collected at discharge. The analytical data set includes values from 13,455 genes for 130 samples, post filtering. As reported previously, the average number of sequence reads in the samples were high (9.93 ± 3.69 million sequence reads). Overall, approximately 60% of possible genes showed detectable transcript as expected for a subset of differentiated cell type (16) as shown in **Supplemental Figure 2**.

### Molecular Markers for BPD

**Figure 1:**
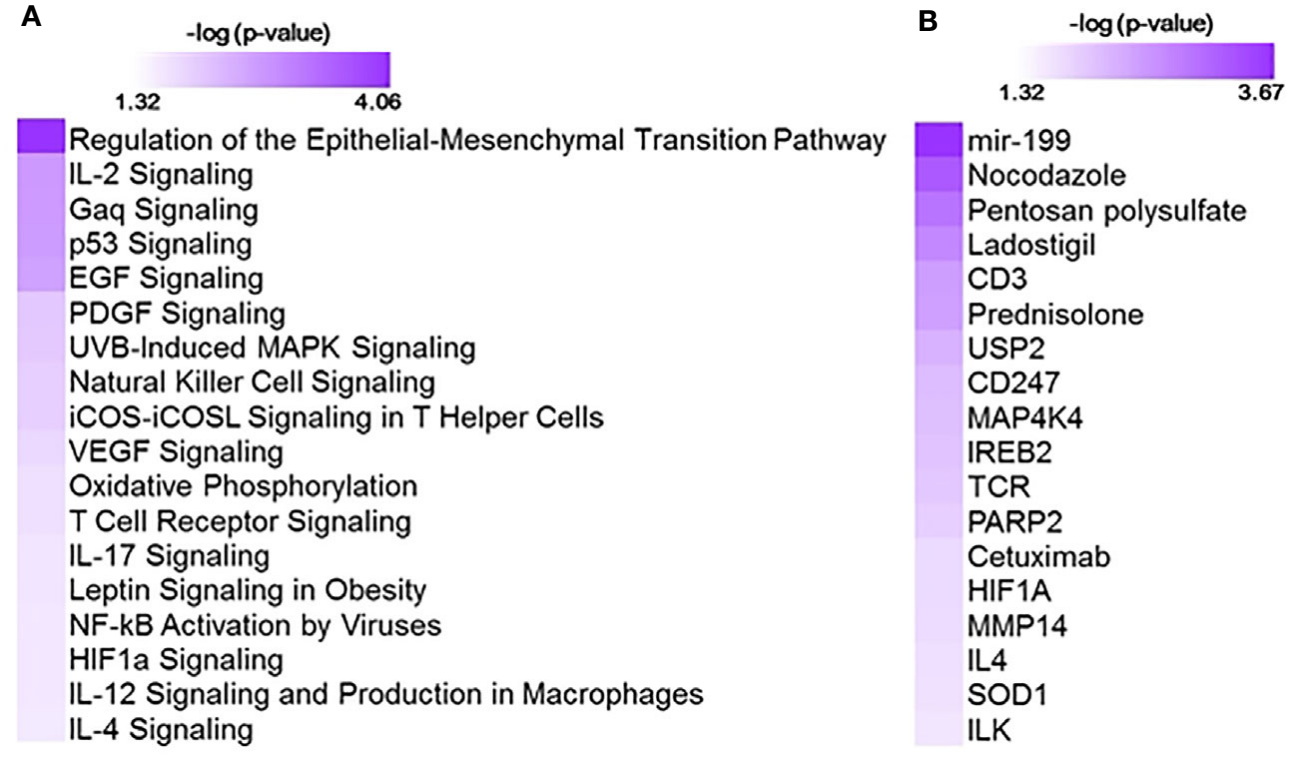
Functional analysis using 501 genes associated with oxygen utilization at day 14 (Oxygen_AUC14_) identified with 104 pathways and 300 upstream regulators. Shown here are selected significant canonical pathways **(A)** and upstream regulators **(B)**, along with their significance level (-logP) as generated by Ingenuity Pathway Analysis (IPA).

Gene expression patterns associated with cumulative oxygen exposure (over the first 14 days of life) in subjects at greatest risk for BPD (born at GAB<29 weeks; n=72) was assessed. Rank correlation analysis identified 501 genes to be significantly associated (at pFDR<0.1) with oxygen exposure, of which 403 were upregulated in BPD, while 98 genes were downregulated in BPD. The magnitude of change, however, was not large, with only 1 gene (*GPCPD1*) induced by 2.3 fold, while all other changes were less than 2-fold, irrespective of the directionality. Twelve of these genes (*RETN, EPHX2, CD27, NOSIP, APOA1BP, TMCO6, KLHL3, B3GALNT1, SLC9A4, PRKCD, ZNF791, and B3GNT2*) were also identified as differentially expressed in BPD in an independent study studying BPD markers in PBMCs (15). The 501 genes, when further assessed for functional classification by Ingenuity Pathway Analysis (IPA), were found to be associated with 104 pathways and 300 upstream regulators (**Figure 1**). The pathways associated with oxygen utilization included TGFB signaling (epithelial-mesenchymal transition), and multiple immune signaling pathways, while tubule formation associated and immunologic molecules were present among the upstream regulators.

**Figure 2:**
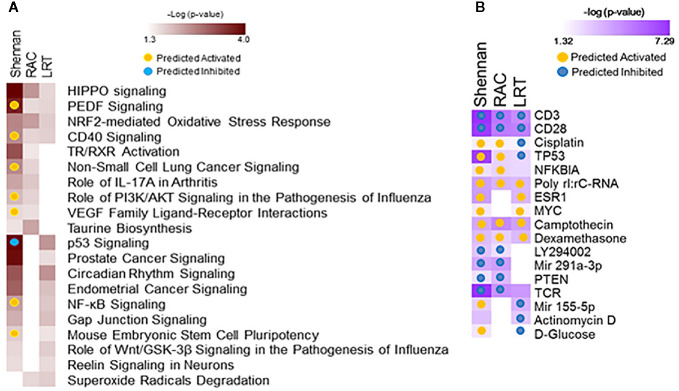
Functional analysis of genes associated with bronchopulmonary dysplasia (BPD). Separate analyses were performed for gene sets identified from analysis of 571 genes associated with BPD (Shennan) and 101 genes associated with room-air challenge (RAC) as identified by SAM-Seq, and 92 genes identified by Screened Principal Components Analysis (sPCA) [adjusted for gestational age at birth (GAB)] to be associated with BPD (Shennan). Selected canonical pathways **(A)** and upstream regulators **(B)** identified are listed for each analysis (columns), with significance (-logP) and directionality (activated/inhibited) as generated by Ingenuity Pathway Analysis (IPA).

Data from 72 subjects born at <29 weeks CGA was used to identify gene expression associated with BPD as defined by physiologic challenge (RAC) or by Shennan criteria. Using SAM-Seq (at mFDR<0.1), 571 genes were differentially expressed in subjects receiving a diagnosis of BPD (Shennan). While all of the 571 genes were upregulated in BPD, only five genes (*GPCPD1, TMEM2, USP2, TSPYL2, and ELL2*) showed a magnitude of induction of greater than 2.0 fold. Fourteen of these genes (*RNF125, FEM1C, FAM54A, ZNF791, AXIN2, B3GNT2, ZNF565, SPON1, TIPARP, ZBTB3, FAM115C, PELO, MXD1, and PFKFB3*) were also identified as differentially expressed, and over expressed in BPD in an independent study looking at BPD markers in PBMCs (15). These 571 genes, when further assessed for functional classification by IPA, identified 113 canonical pathways and 409 upstream regulators to be associated with BPD. In addition, 105 genes were differentially expressed (SAM-Seq at mFDR<0.1) in subjects who failed a room air challenge. While all of the 105 genes were upregulated in BPD, only two genes (*GPCPD1 and TSPYL2*) showed a magnitude of induction of greater than 2.0 fold. These 105 genes when analyzed by IPA, identified 18 canonical pathways and 415 upstream regulators (**Figure 2**). Among the pathways associated with BPD, *PEDF, CD40, PI3K/AKT, VEGF* and *NF-kB* signaling were predicted to be activated, while p53 signaling was inhibited in BPD. Among the BPD associated upstream regulators, *CD23, CD28, PTEN*, and *TCR*, are inhibited, while *NFκB* inhibitor, *camptothecin*, and *dexamethasone* were activated in BPD.

**Figure 3:**
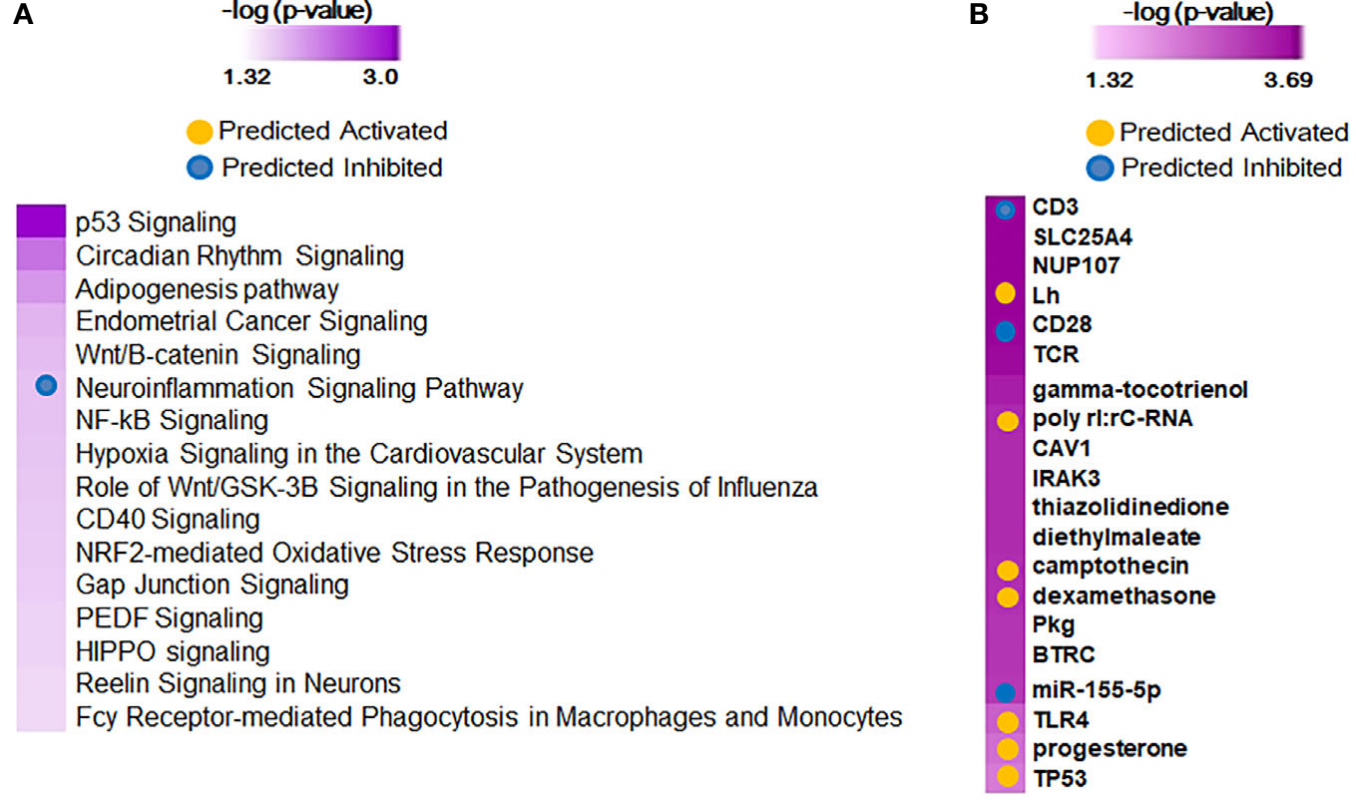
Functional analysis of 75 predictor genes for bronchopulmonary dysplasia (BPD) defined by Likelihood Ratio Test (LRT), identified 113 canonical pathways and 409 upstream regulators. Selected significant canonical pathways **(A)** and upstream regulators **(B)** identified are shown, along with their significance level (-logP) as generated by Ingenuity Pathway Analysis (IPA).

When adjusted for gestational age at birth, 75 genes were associated with the diagnosis of BPD (Shennan) as identified by the Likelihood Ratio Test (LRT at FDR<0.1), and were upregulated in BPD. On further analysis by IPA, the genes provided 113 canonical pathways and 409 upstream regulators (**Figure 3**), of which neuroinflammation pathway appeared to be inhibited, while upstream regulators, *CD23, CD28*, and *TCR*, are inhibited, and *camptothecin*, and *dexamethasone* were activated in BPD. Screened Principal Components Analysis (sPCA), with screening adjusted for GAB, outperformed both screened LASSO and forward selection and identified a classifier gene set consisting of 92 genes (naïve AUC=0.86; CV-AUC=0.71) associated with BPD (Shennan), representing 21 canonical pathways and 253 upstream regulators (**Supplemental Figure 3**). All of the 92 genes were upregulated in BPD, however, none of the genes except *GPCPD1* had a magnitude of induction of greater than two-fold. These 92 genes were also inclusive of all the 75 genes identified by LRT. We subsequently assessed gene expression changes in BPD based on multiple physiologic and clinical definitions and were successful in identifying nine genes (*GPCPD1, MTSS1L, USP15, DDX24, KLF9, CLK1, ZC3H7A, ITCH*, and *PIK3R1*) that were consistently different, and upregulated in BPD, irrespective of definitions, or analytical approaches (**Supplemental Figure 4**).

### Classifiers for PRD Status

**Figure 4:**
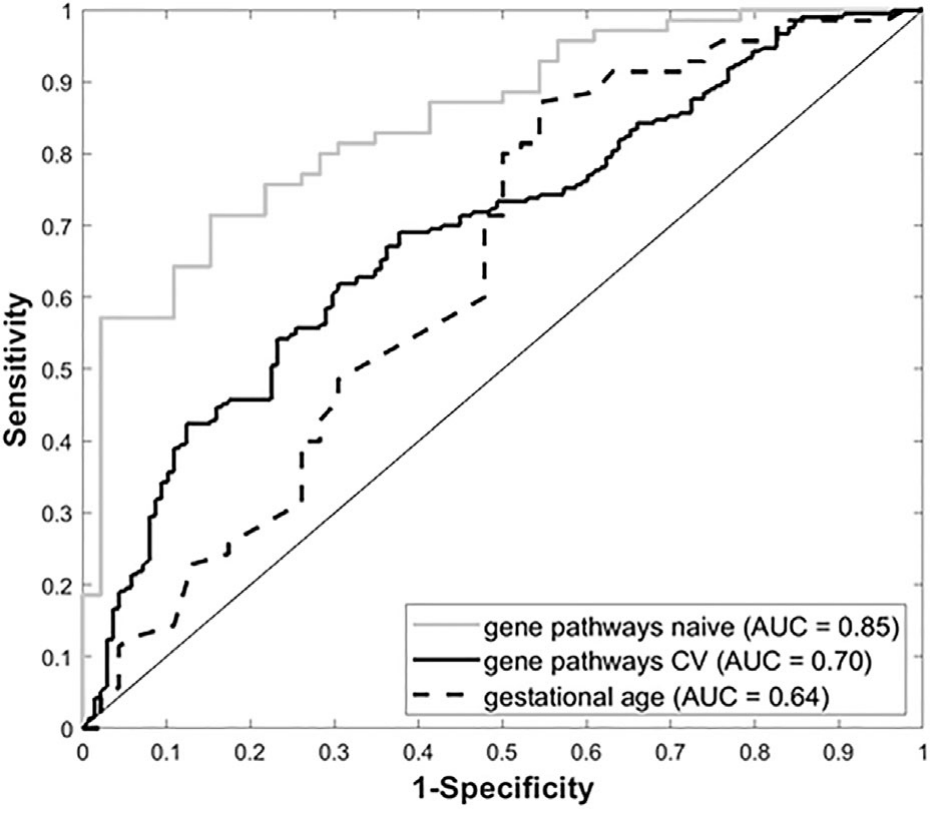
Receiver Operating Characteristic (ROC) curves for gestational age and our four-pathway 28-gene model, with associated Area Under the ROC Curve (AUC).

Gene expression data from all subjects with PRD status (n=116), was used to identify a set of marker genes, based on our novel *canonical pathway analysis* (45), in order to classify the subjects by PRD status (PRD: n=70 and No PRD: n=46). Screened logistic forward selection outperformed both screened LASSO and sPCA, and gestational age was excluded since it did not improve performance. This process identified a set of 28 genes, derived from four canonical pathways (**Table 2**), which predicted PRD status with a moderately high degree of accuracy (naïve AUC=0.85; CV-AUC=0.70) (**Figure 4**). Interestingly, gene predictors of PRD were associated with pathways involving *TGFB* signaling, and organic ion transport. Several genes identified are also involved in T cell skewing and activation (e.g., *PRKCZ* and *FKBP1A*).

**Table 2:** Canonical pathway based analysis selected four pathways including a total of 28 genes using cross-validated screened logistic forward selection for prediction of Post-Prematurity Respiratory Disease (PRD) status.

Source: Pathway	Pathway Coefficient (logOR)	Gene	Gene OR
**Reactome: Recycling Of Bile Acids And Salts**	-1.49	SLC10A1	0.61
		SLC27A5	0.66
		SLCO1A2	0.71
**Reactome: Transport Of Organic Anions**	1.71	SLCO1A2	0.71
		SLCO3A1	1.27
		SLCO4A1	1.58
		SLCO4C1	1.45
**Reactome: TGF-Beta Receptor Signaling In Epithelial To Mesenchyme Transition**	-0.49	ARHGEF18	0.93
		CGN	0.84
		F11R	0.77
		FKBP1A	0.72
		PARD3	0.58
		PARD6A	0.88
		PRKCZ	0.69
		RHOA	0.76
		RPS27A	0.85
		SMURF1	0.91
		TGFB1	1.07
		TGFBR1	0.81
		TGFBR2	1.09
		UBA52	0.87
**Biocarta: Cell2Cell Pathway**	-0.46	ACTN1	0.5
		CSK	0.86
		CTNNA1	0.87
		CTNNB1	1.01
		PECAM1	0.65
		PTK2	0.76
		PXN	0.76
		VCL	0.82

*Pathway logOR are log of odds ratios per standard deviation (SD) of the 1st PC of the screened genes (Wilcoxon p<0.1) within the pathway. *** Gene OR = exp (Gene Loading * log (Screened Pathway OR)/SDPathway) = (Pathway OR)(Gene Loading/(SD of Pathway)).

Shown are pathway and gene names, estimated pathway coefficients (logOR), and constrained gene odds ratios (OR) factoring in the PCA loadings for each gene within a pathway.

### qPCR Validation

Molecular validation of the BPD-associated transcriptomic changes, was attempted on a set of eleven genes selected based on their magnitude of difference or biological relevance, by quantitative reverse transcriptase-polymerase chain reaction (qPCR) (**Table 3**). For each gene, the UQ normalized RNA-seq counts were correlated with the gene expression levels determined by qPCR of the CD8+ T cell cDNA as defined by the dCt. *GAPDH* was used as the endogenous control or housekeeping gene, whose Ct was subtracted from gene Ct to determine the dCT values for each of the genes tested. Spearman’s rank correlation coefficient was estimated along with an associated p-value for each gene. We observed validation of the sequence data for nine of the 11 genes, as defined by a significant Spearman rank correlation (p<0.05) in expression between sequence-based and qPCR-based expression levels. Three genes (*KLF9, DLG5*, *and ZNF44*) differed in expression between BPD and non-BPD subjects (p<0.05), while one additional gene (*PSME4*) had borderline yet insufficient evidence of a difference (0.05<p<0.10).

**Table 3:** Validation of bronchopulmonary dysplasia (BPD) markers.

Gene	Rank Correlation (r, GAPDH)	P-Value
*USP15*	0.49	<0.01
*SOD2*	0.35	<0.01
*ITCH*	0.13	0.27
*AHR*	0.28	0.02
PSME4**	0.34	<0.1
*STAT1*	0.60	<0.01
*GFI1*	0.18	0.14
KLF9*	0.29	0.02
DLG5*	0.27	0.02
ZNF44*	0.25	0.04
*NEAT1*	0.42	<0.01

*KLF9, DLG5, and ZNF44 differed in expression between BPD and non-BPD subjects (p<0.05),

**PSME4 had borderline yet insufficient evidence of a difference between BPD and non-BPD (0.05<p<0.10).

## Discussion

Premature birth is defined as birth taking place prior to 37 weeks of GAB. Prematurity associated lung diseases have been reported to affect not only children as newborns but to also predispose to prolonged respiratory morbidity later in life. Unfortunately, not much is known about the pathophysiology of the prematurity associated lung diseases, such as BPD, and other chronic and prolonged childhood respiratory diseases. BPD is a complex disorder involving genetic–environmental interactions, with each preterm subject having a range (e.g., 1%–99%) of both hereditary and environmental risks (46). We have previously published data on hereditary components to BPD risk by genetic analysis in the PROP cohort, including some of the subjects described in the current manuscript (47, 48). Transcriptomic assessment using gene-expression microarrays has previously been used to identify markers for normal lung development as well as BPD (14, 49, 50). As an alternative to lung tissues, gene expression analyses using peripheral blood have been used in lung diseases to study pathogenesis, severity, and recently as a diagnostic tool (45, 51). In this study, we have used high-throughput sequencing to explore peripheral gene-expression changes associated with prematurity and helps to add to the literature base on potential defects in the immune system of infants with BPD (11). Our analysis identified 571 genes differentially expressed in subjects with diagnosed instances of BPD when compared to extremely low birth weight (ELBW) controls born at less than 29 weeks of GAB by SAM-Seq. An independent study also that examined BPD marker genes from bulk PBMCs identified 12 genes (*RETN, EPHX2, CD27, NOSIP, APOA1BP, TMCO6, KLHL3, B3GALNT1, SLC9A4, PRKCD, ZNF791*, and *B3GNT2*) that were differentially expressed in BPD (15). Thus, our results are consistent with published literature.

Our gene list is further restricted to 92, when we adjust for gestational age at birth (sPCA) as shown in **Supplemental Figure 4**. In addition, we have identified markers, pathways and upstream regulators putatively associated with cumulative oxygen utilization. The pathway most closely associated with oxygen utilization involves several *TGFB* signaling genes (epithelial-mesenchymal transition pathway), which is important for helping to diminish CD8+ T cell activation (28–32). Additional pathways involved with T cell activation (e.g., T cell receptor signaling) and differentiation (e.g., IL-12 signaling). Thus, we have identified pathways that could be useful for identifying new therapeutic targets to treat the postnatal inflammation of preterm infants and to improve the health of children with BPD (52).

Among the differentially expressed genes associated with BPD, the *PFKFB3* gene was not only consistently identified as differentially expressed in BPD subjects irrespective of the approach used, but was also identified as a BPD marker in an independent transcriptomic analysis of PBMCs derived from BPD subjects (15). Through murine studies *PFKFB3* has been identified as potential therapeutic target for the treatment of Pulmonary Hypertension (PH) (53). Increased expression *PFKFB3* in BPD is consistent with PH associated with BPD which is characterized by abnormal vascular remodeling, and vascular growth arrest, which are well documented pathophysiology associated with BPD (54). *In vitro* studies have reported that *PFKFB3-kinase* activity attenuates the activation of T cells, and demonstrated the effectiveness of *PFKFB3* antagonists, even in small amounts, as T cell immunosuppressive agents (55). One of the differentially expressed genes, *KLF9*, has been previously identified as differentially expressed in T cells from patients with autoimmune rheumatoid arthritis (56), while another, *DLG5*, is involved in the *HIPPO* pathway and modulated *TGFB* signaling (57–59).

Pathway analyses indicate dysregulation of *NRF2, HIPPO and CD40* pathways to be consistently associated with BPD. Another marker, *PSME4*, has been associated with tuberculosis through *in vitro* and *in vivo* cultures, and is also related to *CD4, IFNβ1*, and *TGFBI* pathways (60). Interestingly, *NRF2* has been linked to the generation of reactive oxygen species that contributes to inflammation in a variety of diseases (61). The *HIPPO* has been shown to play a role in T cell receptor signaling and in Th17 differentiation (62, 63). Systemic administration of agonistic *CD40* antibody has been shown to increase CD8+ T cell responses in the lungs of non-human primates (64). While *IL2* signaling is expected, *Gnaq* is known to play a role in survival of immune cells (B and T-cells) (65, 66). In addition, *iCOS-iCOSL* are known to be involved in T-cell skewing, and reduced SOD expression is related to impaired CD8+ T cell responses in tumor infiltrating lymphocytes (67). Interestingly, regulators associated by anti-survival, dexamethasone and camptothecin, appear to be activated in BPD, while T cell co-receptors, *CD3* and *CD28*, appear to be inhibited in BPD. One study shows that T cells isolated from patients with chronic viral infection rely on topoisomerase activity to maintain DNA stability and inhibit apoptosis (68). It is well established that chronically activated T cells become hyporesponsive to T cell receptor mediated stimulation (69). These may indicate potential arrest in lung development as a consequence of BPD and indicate induced immune and stress response as a result of therapeutic responses to BPD, due to either oxidative stress, or surfactant treatment (70). Thus, we have identified significant differences in the expression of several genes and pathways in BPD subjects that are related to T cell signaling and effector function.

In addition to studying CD8+ T cell gene expression in relationship to BPD, we also explored multiple approaches to identify a set of genes whose expression may be useful for classification of markers associated with Post-Prematurity Respiratory Disease (PRD) respiratory morbidity. This included approaches to leverage biological priors as a means of identifying the most robust predictors since (1) expression changes at the individual gene level alone may not be sufficient to identify biologically meaningful data and (2) there exists substantial statistical advantages to dimension reduction strategies in the analysis of genome-wide data. We used a curated list of genes (71) to partition our transcriptomic data into 1,330 biologically-relevant gene sets. Using a novel data reduction approach, we identified a 28 gene set classifier that groups subjects according to their PRD status with a moderately high degree of accuracy. Similar to BPD, the *TGFB* pathway was also found to be associated with PRD status. Several genes identified control T cell function. For instance, *SMURF1* accumulation in cells infected with RNA viruses leads to the downregulation of Type I Interferons (72). *RhoA* is a *GTPase* plays an essential role in the migration and activation of T cells (73). *PRKCZ* is a protein kinase C family member that is highly expressed in Th2 CD4+ T cells (74). *PARD3* and *PARD6A* have been shown regulate the *RhoA* signaling pathway (75). *PARD3* also modulates *HIPPO* signaling pathways (75). *FKBP1A* is a signaling molecule in the *mTOR* pathway that regulates memory CD8+ T cell formation (76). Expression of *F11Rm* which encodes for the protein junctional adhesion molecule A, plays a role in T cell adhesion and migration that is upregulated in T cells of lupus patients **(** 77 **).** Their association with persistent respiratory disease indicates propensity to future immunological complications, which may result in chronic lung disorders. Even with the limited set of predictive markers, it does appear that gene expression changes, in peripheral blood at the time of initial discharge after birth, are indicative of future respiratory diseases later in childhood.

One of the limitations of this study is the use of CD8+ T-cells in identifying biomarkers of a lung disease. While peripheral markers have been widely used in identifying expression based markers, it needs to be acknowledged that these hematopoietic cells are from different cell lineage from the pulmonary system, which is the primary organ system affected in BPD and subsequently in PRD. However, even with only one cell type of a different lineage, we have been able to identify previously known, as well as novel molecular markers, and pathways, associated with pulmonary disease due to premature birth. In a longitudinal study involving a subset of subjects from the current cohort, we were able to identify differences in T cell development, post birth, which were able to predict respiratory outcome at 1 year of age (25). Another limitation of the study is the usage of multiple definitions of BPD in order to assess transcriptomic changes caused by it. Despite the various definitions and analytical approaches used, we were able to identify a set of nine marker genes that were observed to be increased in BPD, irrespective of the diagnostic definition and approach used. By using approaches similar to those presented here, researchers will be able to develop better correlations between clinical courses of preterm infants with alterations in the immune system. Previously published data indicate that the patterns of cytokines in the blood of BPD patients relates to the subtype of disease (19). Through the current study we have established proof-of-principle that gene expression provides value for predicting respiratory morbidity following pre-term birth.

In conclusion, we have successfully generated genome-wide transcriptomic data from sort-purified peripheral CD8+ T cells obtained from early pre-term, late preterm, and term infants. We have identified molecular markers, pathways and upstream regulators putatively associated with cumulative oxygen utilization, BPD diagnosis, and PRD prediction. Further studies are needed to determine if the findings are unique to the circulating T cells sampled in this study, or reflective of similar effects in other cells including lung parenchymal cells.

## Data Availability Statement

The datasets presented in this study can be found in dbGap (https://www.ncbi.nlm.nih.gov/gap/) with accession number phs001297.v1.p1.

## Ethics Statement

The studies involving human participants were reviewed and approved by the (Institutional Review Boards of the University of Rochester (IRB# 00037933) and the Children’s Hospital of Buffalo (IRB# 612707). Written informed consent to participate in this study was provided by the participants’ legal guardian/next of kin.

## Author Contributions

SB, JM, AB, and DP analyzed the data. SB, RM, and DP wrote the paper. RR, AR, GP, and TM conceived and designed the study. RM, TM, and GP oversaw the project. All authors contributed to the article and approved the submitted version.

## Funding

The Prematurity and Respiratory Outcomes Program (PROP) was supported by National Institutes of Health (NIH); National Heart, Lung, and Blood Institute (NHLBI); and *Eunice Kennedy Shriver* National Institute of Child Health and Human Development (U01 HL101794 [to University of Pennsylvania, B. Schmidt]; U01 HL101456 [to Vanderbilt University, J.L. Aschner]; U01 HL101798 [to University of California San Francisco, P.L. Ballard and R.L. Keller]; U01 HL101813 [to University of Rochester and University at Buffalo, G. Pryhuber, R. Ryan, and T. Mariani]; U01 HL101465 [to Washington University, A. Hamvas and T. Ferkol]; U01 HL101800 [to Cincinnati Children’s Hospital Medical Center, A.H. Jobe and C.A. Chougnet]; and 5R01HL105702 [to Indiana University and Duke University, C.M. Cotton, S.D. Davis, and J.A. Voynow]). This research was conducted through cooperative agreements with NHLBI and in collaboration with the PROP Steering Committee. The project described in this publication was supported by the University of Rochester CTSA award UL1 TR002001 from the National Center for Advancing Translational Sciences of the National Institutes of Health. The content is solely the responsibility of the authors and does not necessarily represent the official views of the National Institutes of Health.

## Conflict of Interest

The authors declare that the research was conducted in the absence of any commercial or financial relationships that could be construed as a potential conflict of interest.

## PROP Investigators

Cincinnati Children’s Hospital Medical Center:

Barbara Alexander, RN, Claire Chougnet, PhD, Tari Gratton, PA, James M. Greenberg, MD, Cathy Grisby, BSN, CCRC, William Hardie, MD, Alan H. Jobe MD, PhD, Beth Koch, BHS, RRT, RPFT, Karen McDowell, MD, Kelly Thornton BS

Washington University:

Pamela Bates, CRT, RPFT, RPSGT, Claudia Cleveland, RRT, Thomas Ferkol, MD, Aaron Hamvas, MD, Julie Hoffmann, RN, Mark R. Holland, PhD, James Kemp, MD, Philip T. Levy, MD, Laura Linneman, RN, Jayne Sicard-Su, RN, Gina Simpson, RRT, CPFT, Gautam K. Singh, MD, Barbara Warner, MD

University of California at San Francisco:

Investigators

Philip L. Ballard, MD, PhD^1^, Roberta A. Ballard, MD^1^, David J. Durand, MD^2^, Eric C. Eichenwald, MD^4^, Roberta L. Keller, MD^1^, Amir M. Khan, MD^4^, Leslie Lusk, MD^1^, Jeffrey D. Merrill, MD^3^, Dennis W. Nielson, MD, PhD^1^, Elizabeth E. Rogers, MD^1^

Research Staff

Jeanette M. Asselin, MS, RRT-NPS^2^, Samantha Balan1, Katrina Burson, RN, BSN^4^, Cheryl Chapin^1^, Erna Josiah-Davis, RN, NP^3^, Carmen Garcia, RN, CCRP^4^, Hart Horneman^1^, Rick Hinojosa, BSRT, RRT, CPFT-NPS^4^, Christopher Johnson, MBA, RRT^4^, Susan Kelley, RRT^1^, Karin L. Knowles^1^, M. Layne Lillie, RN, BSN^4^, Karen Martin, RN^4^, Sarah Martin, RN, BSN^1^, Julie Arldt-McAlister, RN, BSN4, Georgia E. McDavid, RN4, Lori Pacello, RCP2, Shawna Rodgers, RN, BSN^4^, Daniel K. Sperry, RN4,^1^

^1^Department of Pediatrics, University of California San Francisco, San Francisco, CA; ^2^Children’s Hospital and Research Center Oakland, Oakland, CA

^3^Alta Bates Summit Medical Center, Berkeley, CA

^4^University of Texas Health Science Center- Houston, Houston, TX

Vanderbilt University:

Judy Aschner, MD, Amy B Beller BSN, Candice Fike, MD, Scott Guthrie, MD, Tina Hartert, MD, Nathalie Maitre, MD, Paul Moore, MD, Mark O’ Hunt, Theresa J. Rogers, RN, Odessa L. Settles, RN, MSN, CM, Steven Steele, RN, Marshall Summar, MD, Sharon Wadley, BSN, RN, CLS

University of Rochester-University at Buffalo:

Investigators:

Carl D’Angio, MD, Vasanth Kumar, MD, Tom Mariani, PhD, Gloria Pryhuber, MD, Clement Ren, MD, Anne Marie Reynolds, MD, MPH, Rita M. Ryan, MD*, Kristin Scheible, MD, Timothy Stevens, MD, MPH

Technical Staff:

Heidie Huyck, BS, Valerie Lunger, MS

Study Staff:

Shannon Castiglione, RN, Aimee Horan, LPN, Deanna Maffet, RN, Jane O’Donnell, PNP, Michael Sacilowski, MAT, Tanya Scalise, RN, BSN, Elizabeth Werner, MPH, Jason Zayac, BS Respiratory Therapists and Nurses:

Kim Bordeaux, RRT, Pam Brown, RRT, Julia Epping, AAS, RT, Lisa Flattery-Walsh, RRT, Donna Germuga, RRT, CPFT, Nancy Jenks, RN, Mary Platt, RN, Eileen Popplewell, RRT, Sandra Prentice, CRT

*Present address, Case Western Reserve University, Cleveland, OH

Duke University:

Kim Ciccio, RN, C. Michael Cotten, M.D., Kim Fisher, Ph.D., Jack Sharp, M.D., Judith A. Voynow, M.D.*

*Present address, Virginia Commonwealth University

Indiana University:

Charles Clem, RRT, Stephanie Davis, M.D., Susan Gunn, NNP, CCRC, Lauren Jewett, RN, CCRC Brenda Poindexter, M.D., M.S.#

#Present address, University of Cincinnati

Steering Committee Chair:

Lynn M. Taussig, MD, University of Denver

NHLBI Program Officer:

Carol J. Blaisdell, MD

University of Pennsylvania Data Coordinating Center:

Scarlett Bellamy, ScD, Maria Blanco, BS, Denise Cifelli, MS, Sara DeMauro, MD,

Jonas Ellenberg, PhD, Rui Feng, PhD, Melissa Fernando, MPH, Howard Panitch, MD,

Barbara Schmidt, MD, MSc Pamela Shaw, PhD, Ann Tierney, BA, MS
