# Supplementary material for: Lymphocyte-Specific Biomarkers Associated With Preterm Birth and Bronchopulmonary Dysplasia
Source: Front Immunol. 2021 Jan 21;11:563473. doi: 10.3389/fimmu.2020.563473 (PMC7859626; doi:10.3389/fimmu.2020.563473)

Supplemental Information for

Lymphocyte-Specific Biomarkers Associated with Preterm Birth and Bronchopulmonary Dysplasia

[1]Soumyaroop Bhattacharya, [1]Jared Mereness, [2]Andrea Baran, [1]Ravi Misra, [2]Derick Peterson, [3,4]Rita Ryan, [3]Anne Marie Reynolds, [1]Gloria Pryhuber and [1]Thomas Mariani
[1]Division of Neonatology, Department of Pediatrics, and [2]Department of Biostatistics and Computational Biology, University of Rochester, Rochester, NY,
[3]Women & Children's Hospital of Buffalo, Buffalo, NY, and [4] Department of Pediatrics, Medical University of South Carolina, Charleston, SC, USA

Addresses for Correspondence
Thomas J Mariani, PhD
Division of Neonatology and
Pediatric Molecular and Personalized Medicine Program
University of Rochester Medical Center

601 Elmwood Ave, Box 850
Rochester, NY 14642, USA.

Phone: 585-276-4616
E-mail: Tom_Mariani@urmc.rochester.edu.

Gloria S. Pryhuber, MD
Division of Neonatology
University of Rochester Medical Center
Box 651, 601 Elmwood Avenue
Rochester, NY 14642, USA
Phone: 585-273-4120
Email: gloria_pryhuber@urmc.rochester.edu

Supplemental Table 1:

**Definition**	**Criteria**
BPD (Shennan)	Supplemental oxygen requirement at 36 weeks PMA in infants born with birth weight (BW) < 1,500 g [1]
BPD (Physiologic) or Room Air Challenge (RAC)	Requirement of oxygen support (>21%) for at least 28 days and a subsequent assessment at 36 weeks PMA or discharge, whichever comes first. [2]
PRD	PRD was diagnosed if there were positive responses on at least 2 caregiver post-discharge questionnaires to the following: (1) hospitalization for respiratory indication, (2) home respiratory support, (3) respiratory medication administration, and/or (4) respiratory symptoms without a cold.[3]
$Oxygen_{AUC14}$	Calculated by the formula defined in Benaron and Benitz (1994) [4] using information recorded in the daily respiratory flowsheet data (FIO2, respiratory support mode, and applied airway pressure or cannula flow) through the first 28 days of life.[3]

References:

1. Shennan, A.T., et al., *Abnormal pulmonary outcomes in premature infants: prediction from oxygen requirement in the neonatal period.* Pediatrics, 1988. **82**(4): p. 527-32.
2. Jobe, A.H. and E. Bancalari, *Bronchopulmonary dysplasia.* American journal of respiratory and critical care medicine, 2001. **163**(7): p. 1723-1729.
3. Dylag, A.M., et al., *Early Neonatal Oxygen Exposure Predicts Pulmonary Morbidity and Functional Deficits at 1 Year.* J Pediatr, 2020. **223**: p. 20-28 e2.
4. Benaron, D.A. and W.E. Benitz, *Maximizing the stability of oxygen delivered via nasal cannula.* Archives of pediatrics & adolescent medicine, 1994. **148**(3): p. 294-300.

Supplemental Table 1: Demographic and Diagnostic information for 130 subjects data from which have been used in transcriptomic analysis.

Subject ID	Site	Gender	Hispanic Ethnicity	Race	Gest Age Birth (GAB) in Weeks	Days of Life at Sample Collection	Shennan BPD	Physiologic BPD (RAC)	Oxygen Utilization at Day 14	PRD Status
J9028	Rochester	Male	No	White	27.43	86.03	No	No	400.50	Yes
J599B	Rochester	Male	No	White	25.00	95.97	Yes	No	3204.00	Yes
J061C	Rochester	Male	No	White	28.57	50.96	Uncl.	No	22.00	No
J84E4	Rochester	Female	No	AA	27.57	50.96	No	No	99.00	Yes
JBD2A	Rochester	Female	No	AA	27.57	80.01	No	No	39.00	Yes
JAAD7	Rochester	Female	No	White	26.57	80.01	Yes	Yes	2358.50	Yes
J94E8	Rochester	Male	No	AA	24.71	80.01	No	Yes	8124.50	Yes
JD1FD	Rochester	Female	No	AA	23.86	127.96	No	No	3887.00	Yes
J96D6	Rochester	Male	No	White	25.43	161.98	No	No	1936.50	Yes
J00F9	Rochester	Female	No	White	27.71	117.04	No	No	236.00	No
J6B6F	Rochester	Male	No	AA	26.71	118.02	No	No	2413.00	Yes
J94F4	Rochester	Female	No	AA	26.00	112.98	No	Yes	1758.50	No
JF22D	Rochester	Male	No	White	27.14	112.98	Yes	Yes	3257.50	Yes
J9977	Rochester	Female	No	White	27.71	84.98	Uncl.	No	340.50	NA
J8DB7	Rochester	Male	No	White	30.43	95.97	NA	NA	0.00	No
J2211	Rochester	Male	No	Asian	26.14	136.01	No	No	1072.00	No
J835B	Rochester	Male	No	White	25.57	112.98	Yes	Yes	1076.00	Yes
J33AF	Rochester	Male	No	White	25.57	60.97	No	No	1490.50	No
J8CBE	Rochester	Female	No	White	25.57	105.00	No	No	1344.00	No
JA82D	Rochester	Female	No	White	28.00	41.02	Uncl.	No	355.00	Yes
J2B52	Rochester	Female	No	Other	30.14	89.95	NA	NA	188.53	Yes
JB14F	Rochester	Female	No	White	32.00	125.02	NA	NA	4.00	Yes
J4D32	Rochester	Male	No	White	32.00	140.98	NA	NA	112.00	Yes
J80AD	Rochester	Female	No	White	28.86	132.02	No	No	333.50	Yes
J8648	Rochester	Female	No	White	31.29	52.99	NA	NA	170.50	Yes
J1B12	Rochester	Female	No	AA	24.14	124.04	No	No	2951.50	No
J8C76	Rochester	Female	Yes	White	28.00	70.00	No	No	440.50	No
JE573	Rochester	Female	No	AA	23.29	20.02	No	No	11204.50	No
J6BE4	Rochester	Male	No	White	32.14	23.03	NA	NA	0.00	No
JDCAF	Rochester	Female	No	White	32.14	91.00	NA	NA	0.00	No
JEACE	Rochester	Male	No	White	32.14	51.03	NA	NA	664.00	Yes
J1D4E	Rochester	Male	No	White	24.14	50.05	No	Yes	3407.00	Yes
J6213	Rochester	Female	Yes	Other	34.00	49.00	NA	NA	0.00	No
J5633	Rochester	Female	No	White	32.14	105.98	NA	NA	22.50	No
J92FE	Rochester	Female	No	AA	35.14	84.98	NA	NA	37.00	Yes
J5692	Rochester	Male	No	AA	31.57	105.00	NA	NA	339.00	No
NEF69	Rochester	Male	Yes	White	34.71	22.96	NA	NA	0.00	NA

ND6D4	Rochester	Female	No	White	33.29	37.80	NA	NA	0.00	Yes
NE43D	Rochester	Male	No	White	35.29	38.99	NA	NA	0.00	Yes
J99F0	Rochester	Female	No	White	33.00	126.00	NA	NA	0.00	No
J3150	Rochester	Female	No	White	33.57	14.00	NA	NA	0.00	No
J0A30	Rochester	Female	No	White	32.86	21.00	NA	NA	0.00	No
J8154	Rochester	Male	No	White	29.00	14.00	NA	NA	210.50	Yes
N069F	Rochester	Male	Yes	Other	35.00	24.99	NA	NA	1365.00	No
E8990	Rochester	Male	No	White	39.14	12.95	NA	NA	0.00	No
J1081	Rochester	Male	No	White	34.29	22.05	NA	NA	0.00	NA
JEAC5	Rochester	Female	No	White	28.57	15.05	Yes	Yes	2552.50	Yes
N52A3	Rochester	Female	No	AA	26.86	23.03	No	No	1080.00	Yes
N174C	Rochester	Female	No	AA	23.86	56.00	Yes	Yes	12501.50	Yes
N05A1	Rochester	Female	No	White	26.14	63.00	No	No	1861.50	No
ND064	Rochester	Male	No	White	40.29	84.00	NA	NA	0.00	NA
JF17A	Rochester	Male	No	AA	25.86	42.00	No	No	1452.50	NA
NB0D2	Rochester	Female	No	AA	24.71	49.00	No	No	6826.00	Yes
N2FF6	Rochester	Male	No	White	30.57	63.00	NA	NA	0.00	No
NA9BF	Rochester	Male	No	White	30.57	133.00	NA	NA	110.00	NA
N85B5	Rochester	Female	No	White	30.57	124.04	NA	NA	179.00	Yes
NAF88	Rochester	Female	No	White	39.29	85.96	NA	NA	0.00	No
NA54B	Buffalo	Female	No	White	31.29	89.04	NA	NA	0.00	Yes
NF46C	Buffalo	Male	No	White	25.86	175.00	Yes	Yes	8483.00	Yes
N0E97	Buffalo	Female	No	White	26.57	77.00	Yes	Yes	6067.00	Yes
N8C7D	Buffalo	Male	No	White	31.43	77.00	NA	NA	432.92	Yes
N4696	Buffalo	Male	Yes	Other	28.00	45.01	Yes	Uncl.	7650.50	No
ND11C	Buffalo	Male	No	Other	24.71	98.00	Yes	No	17486.50	NA
N8248	Buffalo	Female	No	White	25.43	94.01	Yes	No	6722.50	Yes
ND69A	Buffalo	Male	No	AA	25.43	35.00	No	No	8038.50	Yes
N9B2B	Buffalo	Male	No	White	24.57	80.99	No	Yes	9628.00	Yes
NFBC1	Buffalo	Male	No	White	28.71	128.94	Uncl.	No	1019.50	Yes
N09F5	Buffalo	Male	No	White	28.71	147.98	Yes	Yes	3199.00	Yes
N5FCD	Buffalo	Male	No	Asian	28.57	94.01	No	No	775.00	No
N2F4C	Buffalo	Male	No	Asian	28.57	97.02	No	No	385.50	Yes
N0987	Buffalo	Male	No	White	31.71	143.01	NA	NA	0.00	Yes
N840F	Buffalo	Female	No	AA	25.00	52.99	Yes	Yes	7025.50	No
NDBE8	Buffalo	Male	No	White	32.71	77.98	NA	NA	120.14	Yes
N6ECA	Buffalo	Male	No	White	32.71	64.96	NA	NA	0.00	Yes
NBE6F	Buffalo	Female	No	White	28.14	57.96	Yes	Yes	618.50	Yes
ND48A	Buffalo	Female	No	White	27.43	52.99	No	Yes	304.00	Yes
ND0F8	Buffalo	Female	No	White	27.43	49.98	No	No	1616.50	No
NEA48	Buffalo	Male	No	White	32.14	122.01	NA	NA	0.00	NA
N8220	Buffalo	Male	No	AA	28.14	40.95	Uncl.	No	869.50	No
N81A1	Buffalo	Male	No	AA	28.14	18.97	Uncl.	No	1831.46	No

ND834	Buffalo	Female	No	White	28.71	82.95	Uncl.	No	0.00	Yes
N7E07	Buffalo	Female	No	White	28.43	58.00	Yes	Yes	4139.50	Yes
N6A6C	Buffalo	Female	No	White	26.14	84.00	Yes	No	2799.00	No
NB35B	Buffalo	Male	No	White	26.86	82.04	No	No	123.00	Yes
NF65F	Buffalo	Male	No	White	25.43	15.96	Yes	Yes	3853.00	No
N9ACD	Buffalo	Male	No	AA	26.14	49.00	Yes	Yes	8016.00	Yes
N5688	Buffalo	Female	No	AA	26.14	49.00	Yes	Yes	7685.00	Yes
NEB8C	Buffalo	Male	No	White	31.86	51.94	NA	NA	0.00	No
NB109	Buffalo	Male	No	AA	24.71	91.00	No	No	9626.00	Yes
NC6F1	Buffalo	Male	No	White	34.29	96.00	NA	NA	0.00	NA
NEF4C	Buffalo	Male	No	AA	34.86	96.04	NA	NA	0.00	No
NCCDE	Buffalo	Female	No	White	28.43	100.00	No	No	1735.81	No
NC52C	Buffalo	Female	No	AA	29.29	105.00	NA	NA	0.00	Yes
N477D	Buffalo	Female	No	AA	27.14	105.00	No	Yes	10201.00	Yes
NA845	Buffalo	Female	No	White	27.00	70.00	Yes	Yes	2656.00	No
N0C8F	Buffalo	Male	No	White	28.00	119.00	Yes	Yes	6635.50	Yes
N83DA	Buffalo	Female	No	White	33.86	30.80	NA	NA	0.00	NA
N679A	Buffalo	Male	No	AAA	26.57	49.00	Yes	Uncl.	2406.00	Yes
NC8D9	Buffalo	Female	No	White	28.14	91.00	Yes	Uncl.	2620.00	Yes
N3037	Buffalo	Male	No	White	25.14	84.00	Yes	No	9185.50	No
N51DF	Buffalo	Male	No	White	33.86	98.00	NA	NA	67.50	Yes
NFFFF	Buffalo	Male	No	AA	35.71	98.00	NA	NA	0.00	No
NB735	Buffalo	Female	No	White	33.14	91.00	NA	NA	851.50	No
ND4BE	Buffalo	Male	No	White	33.14	56.00	NA	NA	1369.50	No
NC2D3	Buffalo	Female	No	White	31.00	123.97	NA	NA	123.50	Yes
N9932	Buffalo	Female	No	AA	26.14	91.00	No	No	5172.00	Yes
NDE39	Buffalo	Female	No	White	32.71	143.99	NA	NA	0.00	No
N880B	Buffalo	Female	No	AA	32.00	21.00	NA	NA	0.00	Yes
N7D89	Buffalo	Female	No	AA	32.00	12.95	NA	NA	0.00	Yes
N2001	Buffalo	Male	No	AA	27.71	45.99	No	No	472.00	Yes
N2C5E	Buffalo	Female	No	White	28.71	46.97	Uncl.	Uncl.	421.12	Yes
N7764	Buffalo	Female	Yes	Other	24.00	70.00	Yes	Yes	12359.50	Yes
N70E9	Buffalo	Male	No	White	26.00	75.95	No	No	3743.50	Yes
NB272	Buffalo	Female	Yes	Other	32.86	75.95	NA	NA	0.00	NA
NEC86	Buffalo	Female	Yes	Other	32.86	15.96	NA	NA	0.00	NA
N046E	Buffalo	Male	No	White	25.14	48.02	Yes	Yes	2379.00	Yes
N7982	Buffalo	Male	No	White	25.14	44.03	No	No	3239.50	No
NB903	Buffalo	Female	No	AA	25.43	23.03	No	Yes	8133.00	Yes
N003F	Buffalo	Male	No	AA	25.43	63.98	Yes	Yes	13007.50	Yes
N1CE2	Buffalo	Female	No	AA	25.43	33.04	Yes	Yes	6948.00	Yes
N6703	Buffalo	Male	No	AA	34.43	44.94	NA	NA	0.00	No
ND977	Buffalo	Male	No	AA	35.43	101.01	NA	NA	0.00	NA
NB4EE	Buffalo	Male	No	White	41.29	74.97	NA	NA	0.00	No

N7F32	Buffalo	Male	No	White	41.14	20.02	NA	NA	0.00	Yes
N49C4	Buffalo	Female	No	White	41.14	21.00	NA	NA	0.00	No
N720A	Buffalo	Female	No	White	30.86	129.01	NA	NA	44.50	Yes
N854C	Buffalo	Female	No	White	40.43	82.95	NA	NA	0.00	No
NBF2B	Buffalo	Male	No	AA	34.00	157.01	NA	NA	0.00	Yes
N2749	Buffalo	Male	No	AA	40.57	102.00	NA	NA	0.00	No
N2895	Buffalo	Male	No	White	40.14	154.98	NA	NA	0.00	No

AA = African American
Uncl. = Unclassified

Supplemental Figure 1: The steps involved in the study and the applications used.

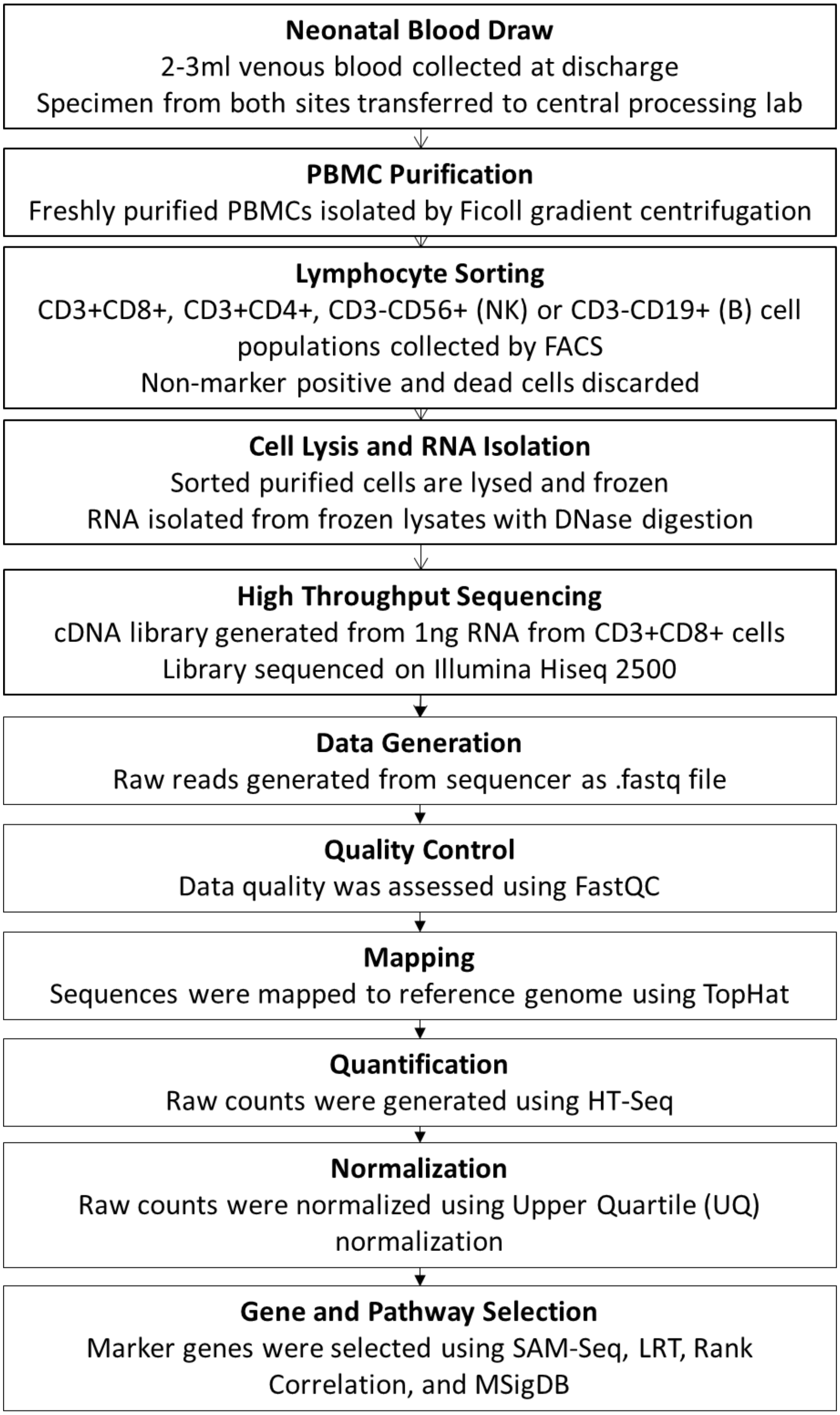

Supplemental Figure 2: RNA-Seq quality statistics: High throughput sequencing was performed using RNA isolated from sorted CD8 + T cells (N=145). The number of input reads (A), the rate of reads mapped (B) and proportion of the genome represented by expressed transcripts (C) for each sample is displayed.

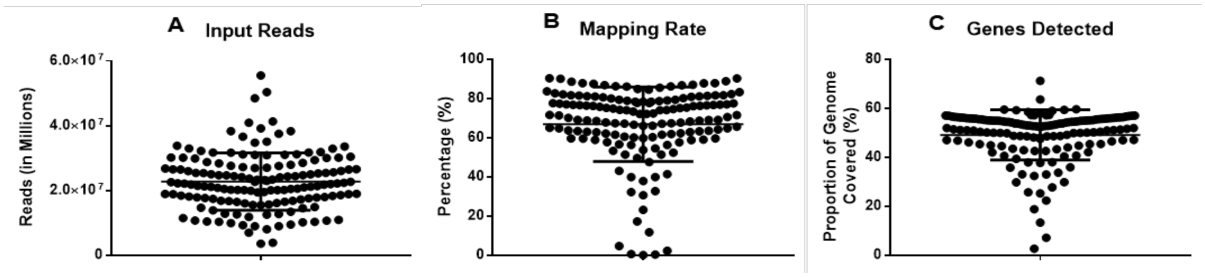

Supplemental Figure 3:   Predictive markers for BPD. A) Normalized expression for 92 genes is shown with subjects in individual columns and genes in individual rows. High expression is indicated by red color and low expression is indicated by green. B) Plots of area under the receiver-operator curve (AUC) comparing the performance of three predictors; using subject gestational age (GA) only, using pathway-based gene expression selected by screened PCA (sPCA) only, or using a combination of GA and sPCA data.

A

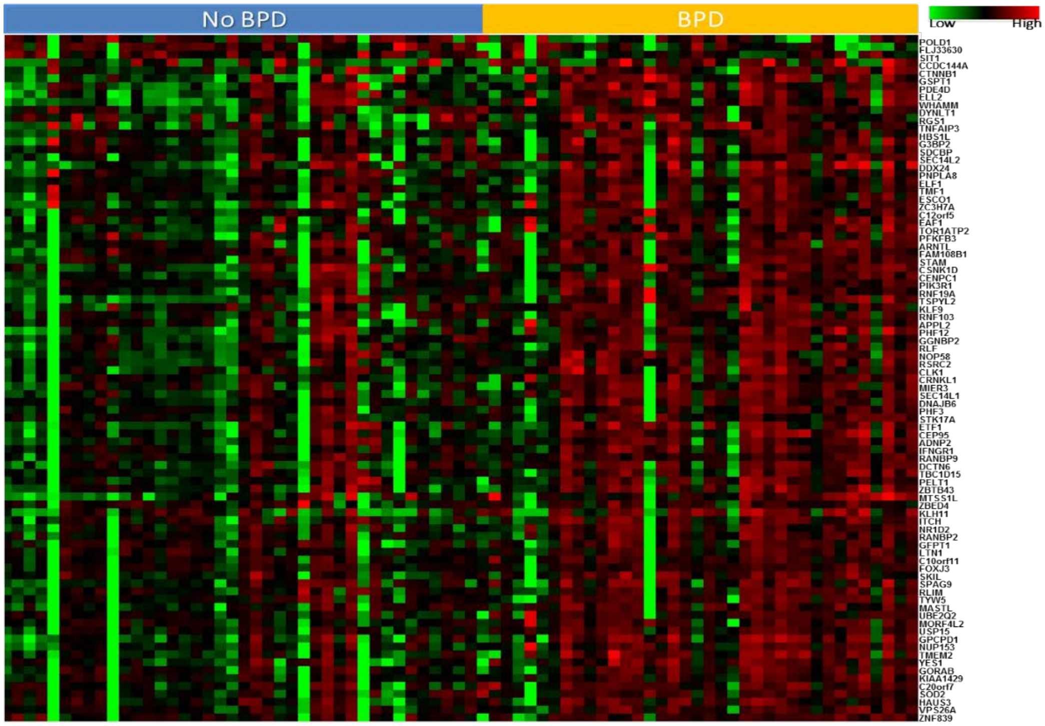

B

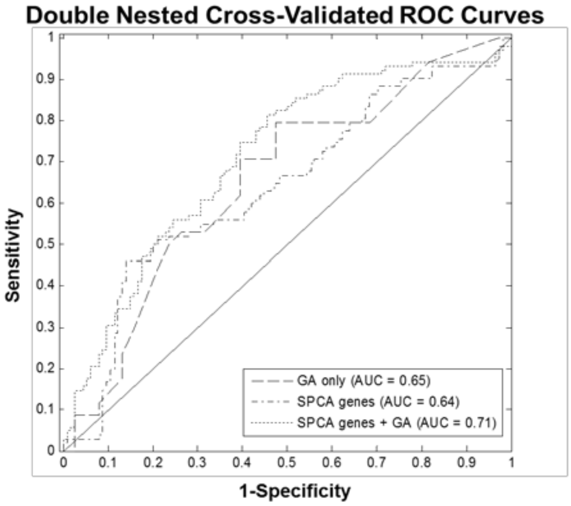

Supplemental Figure 4: Distribution of number of genes differentially expressed in BPD, as assessed by the multiple analytical approaches using the different clinical and physiological definitions of BPD in subjects less than 29 weeks of gestation at the time of birth. O2 (Correlation) indicates correlation analysis with oxygen utilization, RAC (SAM) indicates differentially expressed genes identified by SAM-Seq in BPD as defined by RAC, Shennan (SAM) indicates differentially expressed genes identified by SAM-Seq in BPD as defined by the Shennan definition, and BPD-GAB (sPCA) indicates genes identified as differentially expressed by sPCA in BPD after age adjustment. Irrespective of the method used, or the clinical or physiological definition applied, nine genes were consistently different in BPD subjects.

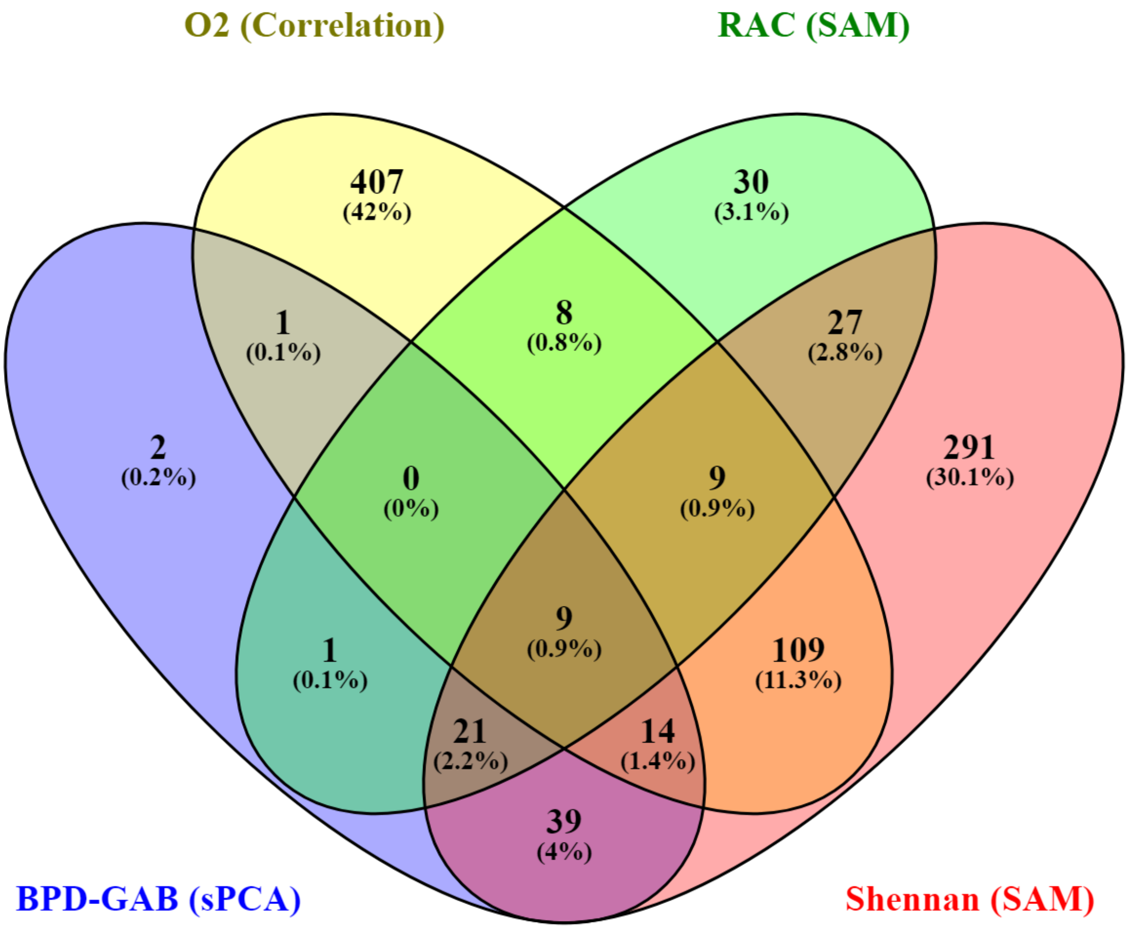

Supplement: Supplementary file 1 [file DataSheet_1.pdf]
